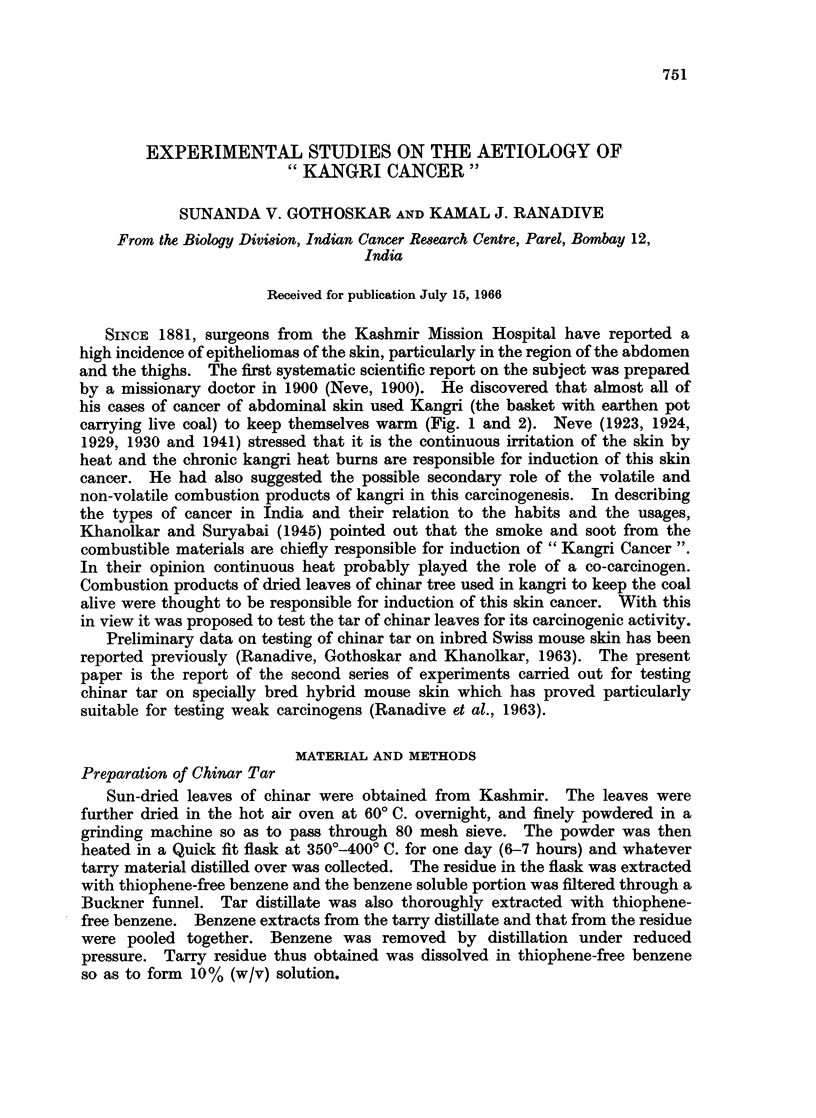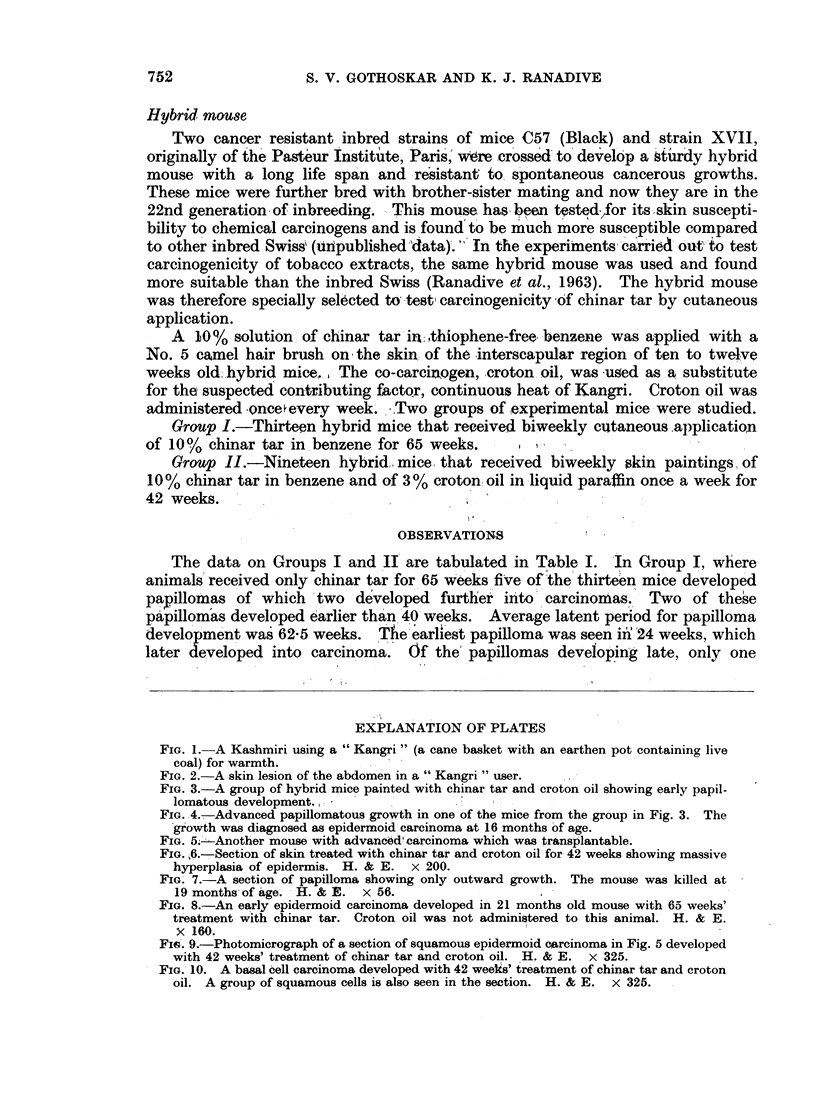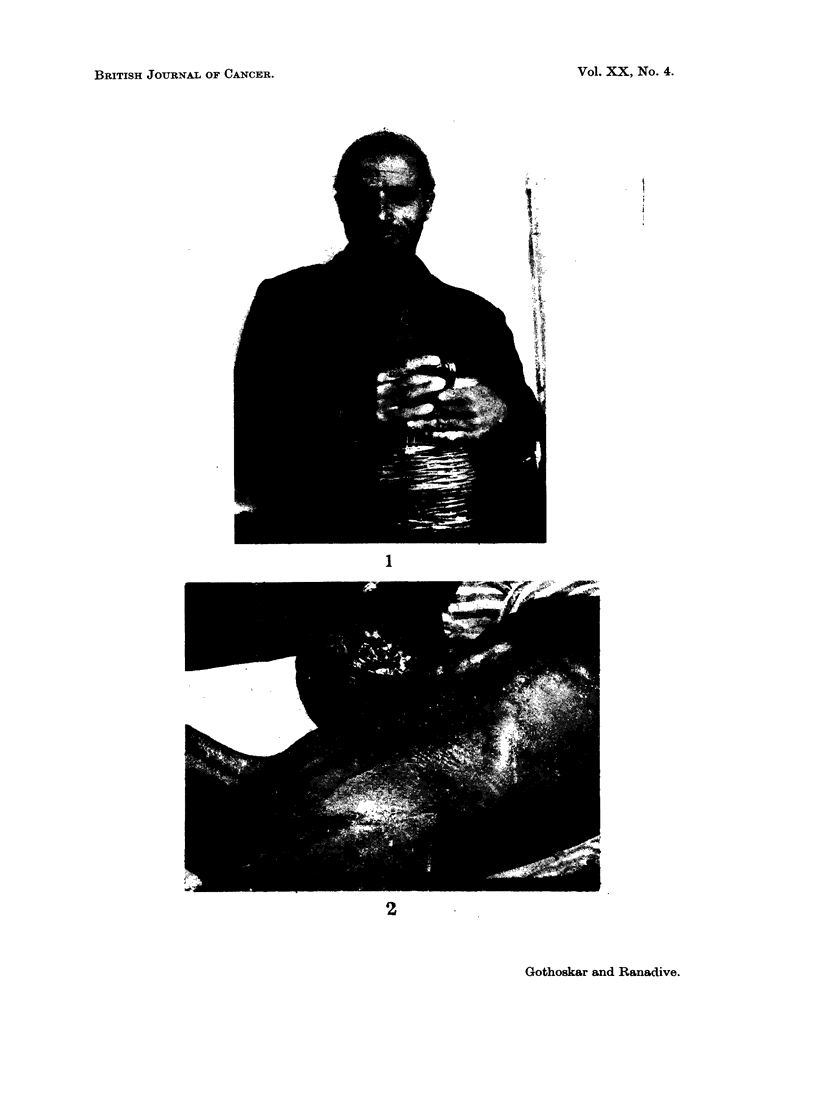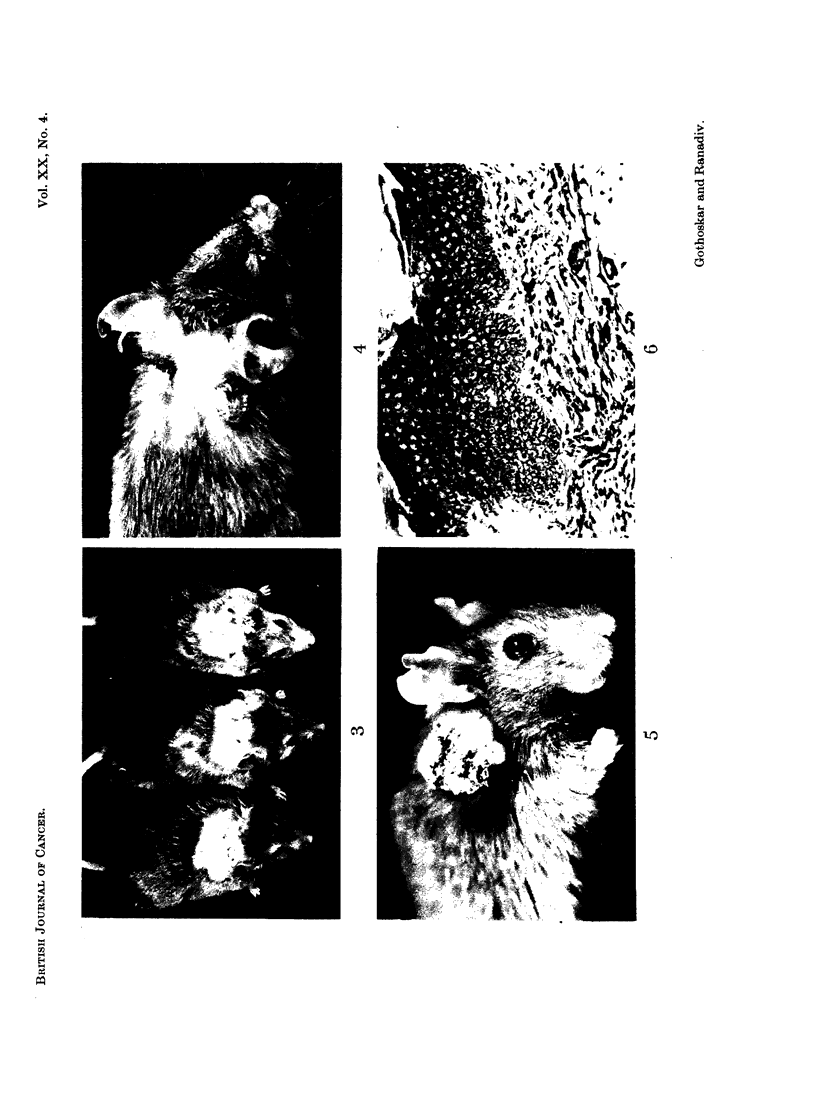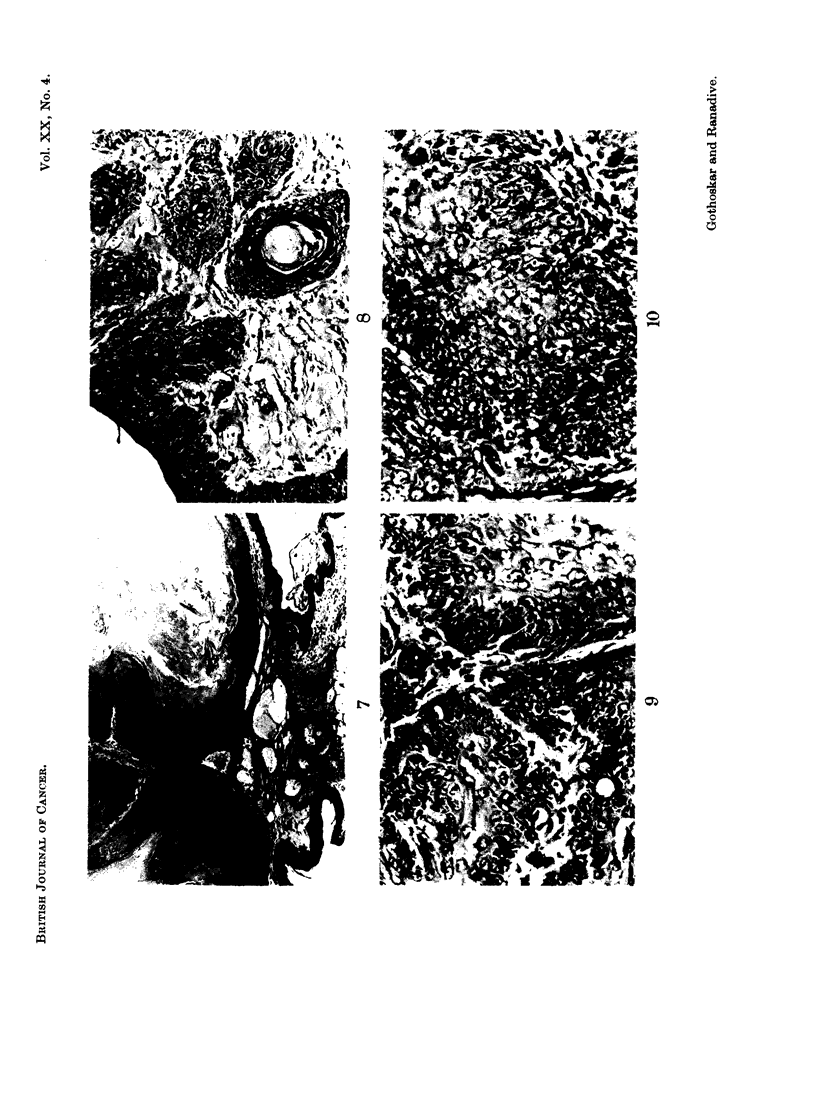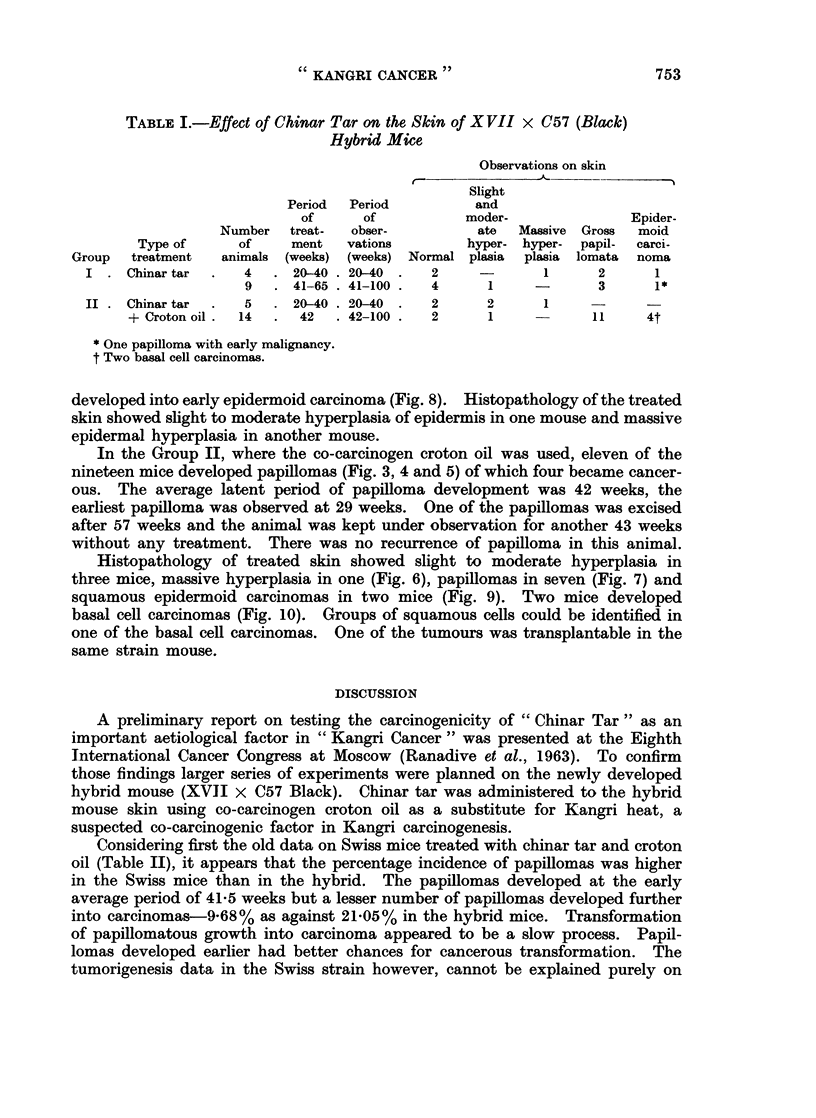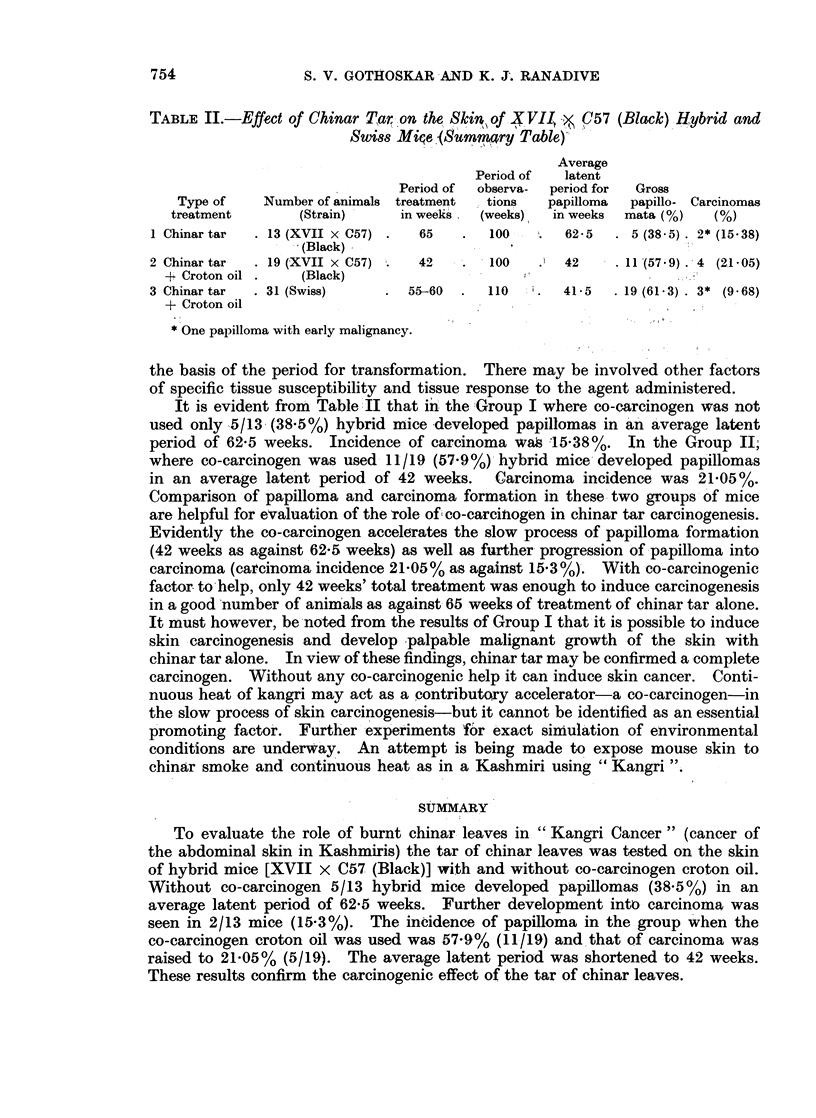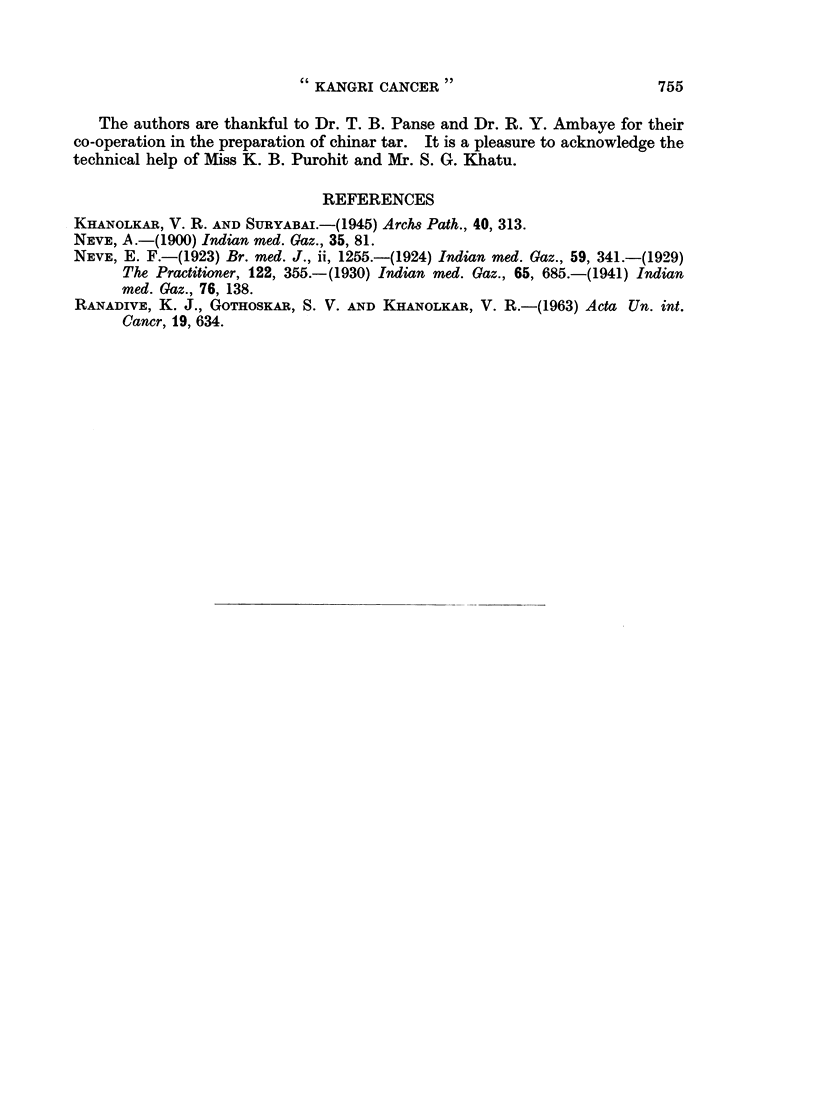# Experimental studies on the aetiology of "Kangri cancer".

**DOI:** 10.1038/bjc.1966.86

**Published:** 1966-12

**Authors:** S. V. Gothoskar, K. J. Ranadive

## Abstract

**Images:**


### EXPERIMENTAL STUDIES ON THE AETIOLOGY OF " KANGRI CANCER "

SUNANDA V. GOTHOSKAR AND KAMAL J. RANADIVE

*From the Biology Division, Indian Cancer Research Centre, Parel, Bombay 12, India*

Received for publication July 15, 1966

SINCE 1881, surgeons from the Kashmir Mission Hospital have reported a high incidence of epitheliomas of the skin, particularly in the region of the abdomen and the thighs. The first systematic scientific report on the subject was prepared by a missionary doctor in 1900 (Neve, 1900). He discovered that almost all of his cases of cancer of abdominal skin used Kangri (the basket with earthen pot carrying live coal) to keep themselves warm (Fig. 1 and 2). Neve (1923, 1924, 1929, 1930 and 1941) stressed that it is the continuous irritation of the skin by heat and the chronic kangri heat burns are responsible for induction of this skin cancer. He had also suggested the possible secondary role of the volatile and non-volatile combustion products of kangri in this carcinogenesis. In describing the types of cancer in India and their relation to the habits and the usages, Khanolkar and Suryabai (1945) pointed out that the smoke and soot from the combustible materials are chiefly responsible for induction of " Kangri Cancer ". In their opinion continuous heat probably played the role of a co-carcinogen. Combustion products of dried leaves of chinar tree used in kangri to keep the coal alive were thought to be responsible for induction of this skin cancer. With this in view it was proposed to test the tar of chinar leaves for its carcinogenic activity.

Preliminary data on testing of chinar tar on inbred Swiss mouse skin has been reported previously (Ranadive, Gothoskar and Khanolkar, 1963). The present paper is the report of the second series of experiments carried out for testing chinar tar on specially bred hybrid mouse skin which has proved particularly suitable for testing weak carcinogens (Ranadive *et al.*, 1963).

### MATERIAL AND METHODS

### *Preparation of Chinar Tar*

Sun-dried leaves of chinar were obtained from Kashmir. The leaves were further dried in the hot air oven at 60° C. overnight, and finely powdered in a grinding machine so as to pass through 80 mesh sieve. The powder was then heated in a Quick fit flask at 350°–400° C. for one day (6–7 hours) and whatever tarry material distilled over was collected. The residue in the flask was extracted with thiophene-free benzene and the benzene soluble portion was filtered through a Buckner funnel. Tar distillate was also thoroughly extracted with thiophene-free benzene. Benzene extracts from the tarry distillate and that from the residue were pooled together. Benzene was removed by distillation under reduced pressure. Tarry residue thus obtained was dissolved in thiophene-free benzene so as to form 10% (w/v) solution.

*Hybrid mouse*

Two cancer resistant inbred strains of mice C57 (Black) and strain XVII, originally of the Pasteur Institute, Paris, were crossed to develop a sturdy hybrid mouse with a long life span and resistant to spontaneous cancerous growths. These mice were further bred with brother-sister mating and now they are in the 22nd generation of inbreeding. This mouse has been tested for its skin susceptibility to chemical carcinogens and is found to be much more susceptible compared to other inbred Swiss (unpublished data). In the experiments carried out to test carcinogenicity of tobacco extracts, the same hybrid mouse was used and found more suitable than the inbred Swiss (Ranadive *et al.*, 1963). The hybrid mouse was therefore specially selected to test carcinogenicity of chinar tar by cutaneous application.

A 10% solution of chinar tar in thiophene-free benzene was applied with a No. 5 camel hair brush on the skin of the interscapular region of ten to twelve weeks old hybrid mice. The co-carcinogen, croton oil, was used as a substitute for the suspected contributing factor, continuous heat of Kangri. Croton oil was administered once every week. Two groups of experimental mice were studied.

*Group I.*—Thirteen hybrid mice that received biweekly cutaneous application of 10% chinar tar in benzene for 65 weeks.

*Group II.*—Nineteen hybrid mice that received biweekly skin paintings of 10% chinar tar in benzene and of 3% croton oil in liquid paraffin once a week for 42 weeks.

### OBSERVATIONS

The data on Groups I and II are tabulated in Table I. In Group I, where animals received only chinar tar for 65 weeks five of the thirteen mice developed papillomas of which two developed further into carcinomas. Two of these papillomas developed earlier than 40 weeks. Average latent period for papilloma development was 62·5 weeks. The earliest papilloma was seen in 24 weeks, which later developed into carcinoma. Of the papillomas developing late, only one

---

### EXPLANATION OF PLATES

FIG. 1.—A Kashmiri using a " Kangri " (a cane basket with an earthen pot containing live coal) for warmth.

FIG. 2.—A skin lesion of the abdomen in a " Kangri " user.

FIG. 3.—A group of hybrid mice painted with chinar tar and croton oil showing early papillomatous development.

FIG. 4.—Advanced papillomatous growth in one of the mice from the group in Fig. 3. The growth was diagnosed as epidermoid carcinoma at 16 months of age.

FIG. 5.—Another mouse with advanced carcinoma which was transplantable.

FIG. 6.—Section of skin treated with chinar tar and croton oil for 42 weeks showing massive hyperplasia of epidermis. H. & E. × 200.

FIG. 7.—A section of papilloma showing only outward growth. The mouse was killed at 19 months of age. H. & E. × 56.

FIG. 8.—An early epidermoid carcinoma developed in 21 months old mouse with 65 weeks' treatment with chinar tar. Croton oil was not administered to this animal. H. & E. × 160.

FIG. 9.—Photomicrograph of a section of squamous epidermoid carcinoma in Fig. 5 developed with 42 weeks' treatment of chinar tar and croton oil. H. & E. × 325.

FIG. 10. A basal cell carcinoma developed with 42 weeks' treatment of chinar tar and croton oil. A group of squamous cells is also seen in the section. H. & E. × 325.

1

2

Gothoskar and Ranadive.

3

4

5

6

Gothoskar and Ranadiv.

7

8

9

10

Gothoskar and Ranadive.

TABLE I.—*Effect of Chinar Tar on the Skin of XVII × C57 (Black) Hybrid Mice*

| | | | | | Observations on skin | | | | |
|---|---|---|---|---|---|---|---|---|---|
| Group | Type of treatment | Number of animals | Period of treatment (weeks) | Period of observations (weeks) | Normal | Slight and moderate hyperplasia | Massive hyperplasia | Gross papillomata | Epidermoid carcinoma |
| I | Chinar tar | 4 | 20–40 | 20–40 | 2 | — | 1 | 2 | 1 |
| | | 9 | 41–65 | 41–100 | 4 | 1 | — | 3 | 1* |
| II | Chinar tar | 5 | 20–40 | 20–40 | 2 | 2 | 1 | — | — |
| | + Croton oil | 14 | 42 | 42–100 | 2 | 1 | — | 11 | 4† |

* One papilloma with early malignancy.
† Two basal cell carcinomas.

developed into early epidermoid carcinoma (Fig. 8). Histopathology of the treated skin showed slight to moderate hyperplasia of epidermis in one mouse and massive epidermal hyperplasia in another mouse.

In the Group II, where the co-carcinogen croton oil was used, eleven of the nineteen mice developed papillomas (Fig. 3, 4 and 5) of which four became cancerous. The average latent period of papilloma development was 42 weeks, the earliest papilloma was observed at 29 weeks. One of the papillomas was excised after 57 weeks and the animal was kept under observation for another 43 weeks without any treatment. There was no recurrence of papilloma in this animal.

Histopathology of treated skin showed slight to moderate hyperplasia in three mice, massive hyperplasia in one (Fig. 6), papillomas in seven (Fig. 7) and squamous epidermoid carcinomas in two mice (Fig. 9). Two mice developed basal cell carcinomas (Fig. 10). Groups of squamous cells could be identified in one of the basal cell carcinomas. One of the tumours was transplantable in the same strain mouse.

### DISCUSSION

A preliminary report on testing the carcinogenicity of " Chinar Tar " as an important aetiological factor in " Kangri Cancer " was presented at the Eighth International Cancer Congress at Moscow (Ranadive *et al.*, 1963). To confirm those findings larger series of experiments were planned on the newly developed hybrid mouse (XVII × C57 Black). Chinar tar was administered to the hybrid mouse skin using co-carcinogen croton oil as a substitute for Kangri heat, a suspected co-carcinogenic factor in Kangri carcinogenesis.

Considering first the old data on Swiss mice treated with chinar tar and croton oil (Table II), it appears that the percentage incidence of papillomas was higher in the Swiss mice than in the hybrid. The papillomas developed at the early average period of 41·5 weeks but a lesser number of papillomas developed further into carcinomas—9·68% as against 21·05% in the hybrid mice. Transformation of papillomatous growth into carcinoma appeared to be a slow process. Papillomas developed earlier had better chances for cancerous transformation. The tumorigenesis data in the Swiss strain however, cannot be explained purely on

TABLE II.—*Effect of Chinar Tar on the Skin of XVII × C57 (Black) Hybrid and Swiss Mice (Summary Table)*

| Type of treatment | Number of animals (Strain) | Period of treatment in weeks | Period of observations (weeks) | Average latent period for papilloma in weeks | Gross papillomata (%) | Carcinomas (%) |
|---|---|---|---|---|---|---|
| 1 Chinar tar | 13 (XVII × C57) (Black) | 65 | 100 | 62·5 | 5 (38·5) | 2* (15·38) |
| 2 Chinar tar + Croton oil | 19 (XVII × C57) (Black) | 42 | 100 | 42 | 11 (57·9) | 4 (21·05) |
| 3 Chinar tar + Croton oil | 31 (Swiss) | 55–60 | 110 | 41·5 | 19 (61·3) | 3* (9·68) |

* One papilloma with early malignancy.

the basis of the period for transformation. There may be involved other factors of specific tissue susceptibility and tissue response to the agent administered.

It is evident from Table II that in the Group I where co-carcinogen was not used only 5/13 (38·5%) hybrid mice developed papillomas in an average latent period of 62·5 weeks. Incidence of carcinoma was 15·38%. In the Group II, where co-carcinogen was used 11/19 (57·9%) hybrid mice developed papillomas in an average latent period of 42 weeks. Carcinoma incidence was 21·05%. Comparison of papilloma and carcinoma formation in these two groups of mice are helpful for evaluation of the role of co-carcinogen in chinar tar carcinogenesis. Evidently the co-carcinogen accelerates the slow process of papilloma formation (42 weeks as against 62·5 weeks) as well as further progression of papilloma into carcinoma (carcinoma incidence 21·05% as against 15·3%). With co-carcinogenic factor to help, only 42 weeks' total treatment was enough to induce carcinogenesis in a good number of animals as against 65 weeks of treatment of chinar tar alone. It must however, be noted from the results of Group I that it is possible to induce skin carcinogenesis and develop palpable malignant growth of the skin with chinar tar alone. In view of these findings, chinar tar may be confirmed a complete carcinogen. Without any co-carcinogenic help it can induce skin cancer. Continuous heat of kangri may act as a contributory accelerator—a co-carcinogen—in the slow process of skin carcinogenesis—but it cannot be identified as an essential promoting factor. Further experiments for exact simulation of environmental conditions are underway. An attempt is being made to expose mouse skin to chinar smoke and continuous heat as in a Kashmiri using " Kangri ".

### SUMMARY

To evaluate the role of burnt chinar leaves in " Kangri Cancer " (cancer of the abdominal skin in Kashmiris) the tar of chinar leaves was tested on the skin of hybrid mice [XVII × C57 (Black)] with and without co-carcinogen croton oil. Without co-carcinogen 5/13 hybrid mice developed papillomas (38·5%) in an average latent period of 62·5 weeks. Further development into carcinoma was seen in 2/13 mice (15·3%). The incidence of papilloma in the group when the co-carcinogen croton oil was used was 57·9% (11/19) and that of carcinoma was raised to 21·05% (5/19). The average latent period was shortened to 42 weeks. These results confirm the carcinogenic effect of the tar of chinar leaves.

The authors are thankful to Dr. T. B. Panse and Dr. R. Y. Ambaye for their co-operation in the preparation of chinar tar. It is a pleasure to acknowledge the technical help of Miss K. B. Purohit and Mr. S. G. Khatu.

### REFERENCES

KHANOLKAR, V. R. AND SURYABAI.—(1945) *Archs Path.*, **40**, 313.

NEVE, A.—(1900) *Indian med. Gaz.*, **35**, 81.

NEVE, E. F.—(1923) *Br. med. J.*, ii, 1255.—(1924) *Indian med. Gaz.*, **59**, 341.—(1929) *The Practitioner*, **122**, 355.—(1930) *Indian med. Gaz.*, **65**, 685.—(1941) *Indian med. Gaz.*, **76**, 138.

RANADIVE, K. J., GOTHOSKAR, S. V. AND KHANOLKAR, V. R.—(1963) *Acta Un. int. Cancr*, **19**, 634.